# *HTRA1*-Related Cerebral Small Vessel Disease: A Review of the Literature

**DOI:** 10.3389/fneur.2020.00545

**Published:** 2020-07-03

**Authors:** Masahiro Uemura, Hiroaki Nozaki, Taisuke Kato, Akihide Koyama, Naoko Sakai, Shoichiro Ando, Masato Kanazawa, Nozomi Hishikawa, Yoshinori Nishimoto, Kiran Polavarapu, Atchayaram Nalini, Akira Hanazono, Daisuke Kuzume, Akihiro Shindo, Mohammad El-Ghanem, Arata Abe, Aki Sato, Mari Yoshida, Takeshi Ikeuchi, Ikuko Mizuta, Toshiki Mizuno, Osamu Onodera

**Affiliations:** ^1^Department of Neurology, Brain Research Institute, Niigata University, Niigata, Japan; ^2^Department of Medical Technology, Graduate School of Health Sciences, Niigata University, Niigata, Japan; ^3^Department of Neurology, Niigata City General Hospital, Niigata, Japan; ^4^Department of System Pathology for Neurological Disorders, Brain Research Institute, Niigata University, Niigata, Japan; ^5^Division of Legal Medicine, Niigata University, Niigata, Japan; ^6^Department of Neurology, Okayama University Graduate School of Medicine, Dentistry and Pharmaceutical Sciences, Okayama, Japan; ^7^Department of Neurology, School of Medicine, Keio University, Tokyo, Japan; ^8^Department of Neurology, National Institute of Mental Health and Neurosciences, Bangalore, India; ^9^Division of Gastroenterology, Hepato-Biliary-Pancreatology and Neurology, Akita University Hospital, Akita, Japan; ^10^Department of Neurology, Chikamori Hospital, Kochi, Japan; ^11^Department of Neurology, Mie University Graduate School of Medicine, Mie, Japan; ^12^Department of Neurology, Neurosurgery and Medical Imaging, University of Arizona-Banner University Medicine, Tucson, AZ, United States; ^13^Department of Neurology, Nippon Medical School Musashi Kosugi Hospital, Kawasaki, Japan; ^14^Department of Neuropathology, Institute for Medical Science of Aging, Aichi Medical University, Nagakute, Japan; ^15^Department of Molecular Genetics, Brain Research Institute, Niigata University, Niigata, Japan; ^16^Department of Neurology, Graduate School of Medical Science, Kyoto Prefectural University of Medicine, Kyoto, Japan

**Keywords:** heritability, vascular dementia, *HTRA1*, carriers, CARASIL

## Abstract

Cerebral autosomal recessive arteriopathy with subcortical infarcts and leukoencephalopathy (CARASIL) is clinically characterized by early-onset dementia, stroke, spondylosis deformans, and alopecia. In CARASIL cases, brain magnetic resonance imaging reveals severe white matter hyperintensities (WMHs), lacunar infarctions, and microbleeds. CARASIL is caused by a homozygous mutation in *high-temperature requirement A serine peptidase 1* (*HTRA1*). Recently, it was reported that several heterozygous mutations in *HTRA1* also cause cerebral small vessel disease (CSVD). Although patients with heterozygous *HTRA1*-related CSVD (symptomatic carriers) are reported to have a milder form of CARASIL, little is known about the clinical and genetic differences between the two diseases. Given this gap in the literature, we collected clinical information on *HTRA1*-related CSVD from a review of the literature to help clarify the differences between symptomatic carriers and CARASIL and the features of both diseases. Forty-six symptomatic carriers and 28 patients with CARASIL were investigated. Twenty-eight mutations in symptomatic carriers and 22 mutations in CARASIL were identified. Missense mutations in symptomatic carriers are more frequently identified in the linker or loop 3 (L3)/loop D (LD) domains, which are critical sites in activating protease activity. The ages at onset of neurological symptoms/signs were significantly higher in symptomatic carriers than in CARASIL, and the frequency of characteristic extraneurological findings and confluent WMHs were significantly higher in CARASIL than in symptomatic carriers. As previously reported, heterozygous *HTRA1*-related CSVD has a milder clinical presentation of CARASIL. It seems that haploinsufficiency can cause CSVD among symptomatic carriers according to the several patients with heterozygous nonsense/frameshift mutations. However, the differing locations of mutations found in the two diseases indicate that distinct molecular mechanisms influence the development of CSVD in patients with *HTRA1*-related CSVD. These findings further support continued careful examination of the pathogenicity of mutations located outside the linker or LD/L3 domain in symptomatic carriers.

## Introduction

Cerebral autosomal recessive arteriopathy with subcortical infarcts and leukoencephalopathy (CARASIL, OMIM 600142) is a hereditary cerebral small vessel disease (CSVD) caused by biallelic loss-of-function mutations in *high-temperature requirement A serine peptidase 1* (*HTRA1*), which upregulates the transforming growth factor β1 (TGF-β1) signal (1). CARASIL is characterized by dementia, stroke, alopecia, and lumbago or spondylosis deformans. On brain magnetic resonance imaging (MRI), severe leukoencephalopathy with multiple lacunar infarctions (LIs), microbleeds (MBs), and brain atrophy are common. Although CARASIL patients were initially reported in Japan, after identification of *HTRA1* as a causative gene, more than 25 CARASIL patients were subsequently identified in other countries, including China, Italy, India, and the United States (2–5).

At present, more than 50 symptomatic carriers of *HTRA1* mutations have been reported (6–10). However, most parents of CARASIL patients are asymptomatic (1, 3, 5, 11–17). It thus remains unclear why certain mutations cause CSVD in *HTRA1* carriers. Previously, we reported that either a deficiency in trimerization or an amino-acid mutation located in the loop D (LD) or loop 3 (L3) domain was common in missense *HTRA1* proteins identified in symptomatic carriers (18). We speculated that these mutations in the *HTRA1* gene may inhibit wild-type (WT) protease activity (7). However, not all the mutations have been proofed to fulfill the pathogenicity. In the present study, we reviewed the literature that describes symptomatic carriers and CARASIL to clarify the molecular and clinical features of *HTRA1*-related CSVD.

## Materials and Methods

### Summary of Mutations in Patients With *HTRA1*-Related CSVD

This study was approved by the ethical board of Niigata University. We reviewed PubMed and Google Scholar databases for reports of *HTRA1*-related CSVD using the search terms “HTRA1 mutation” and “CARASIL.” Only reports published prior to September 2019 were included. After reviewing the literature, mutations identified among patients with *HTRA1*-related CSVD were summarized. Reports of a total of 82 patients with *HTRA1*-related CSVD in 31 articles were identified (1–17, 19–32). Twenty-eight of those patients were CARASIL, and the other 54 were heterozygous *HTRA1*-related CSVD symptomatic carriers.

Each mutation was classified by location, affected domain of *HTRA1*, which includes the insulin-like growth factor binding protein (*IGFBP*), Kazal-like, protease, and PDZ-like domains. Furthermore, the protease domain was divided into three groups: LD, L3, and not L3 or LD. LD and L3 are essential domains required for the protease activities of *HTRA1* via intermolecular communication (33, 34). In the present study, LD was defined as the amino acid positions between 283 and 291. L3 was defined as the position of amino acids between 301 and 314 (7). We added one more region, the “linker region,” which was located between the Kazal-like and protease domains (9). We further searched for the minor allele frequencies of each mutation in *HTRA1* using ExAC (Exome Aggregation Consortium) web browser^1^.

To determine the pathogenicity of missense *HTRA1* mutants, *in silico* analyses using PolyPhen-2 (35), SIFT (36), PANTHER (37), and PROVEAN (38) software from the variation effect on protein structure and function platform for drug discovery, informatics, and structural life science (VaProS-PDIS) website^2^ were performed. Pathogenic mutations were defined using the following criteria: (1) three or more *in silico* analysis showed the following result: probably damaging (PolyPhen-2) or deleterious (SIFT, PANTHER, and PROVEAN) or (2) previous *in vitro* assessment of *HTRA1* mutation that indicated decreased protease activity. These rules satisfied the criteria of “likely pathogenic” as stated by the guidelines of the American College of Medical Genetics and Genomics (39). Mutations without pathogenicity were excluded from further analyses. Then, to investigate the difference between the distribution of mutations in symptomatic carriers and CARASIL, we compared the locations of mutations between groups.

Finally, a 3D model of *HTRA1* (PDB ID: 3NZI) was obtained from the RCSB (Research Collaboratory for Structural Bioinformatics) protein data bank^3^. This model was to create images that demonstrated the locations of missense mutations specific to each group using PyMOL software, version 2.3.0 (Schrodinger, LLC, New York, NY, USA)^4^.

### Clinical Assessments of Symptomatic Carriers and CARASIL

Clinical information, such as neurological symptoms and signs, family history, and risk factors, was obtained from the literature and an in-house clinical data set. Patients with CSVD can present with various neurological symptoms and signs. However, we selectively searched for those with a history of stroke, cognitive impairment, and gait disturbance in the present study, which are the cardinal features of CSVD (other clinical symptoms such as vertigo are less specific to CSVD). We further reviewed the literature for patients with a clinical history of migraine, lumbago/spondylosis deformans, and alopecia of younger onset. Alopecia of younger onset was defined as the age at onset of alopecia in individuals aged ≤40 years, according to the Japanese diagnostic criteria of CARASIL^5^. Family history was defined as the positive history of cognitive impairment, stroke, or leukoencephalopathy. We additionally investigated examined patient brain MRIs to detect the severity of white matter hyperintensities (WMHs) and the presence of LIs. If available, the information of T2^*^-weighted image or susceptibility-weighted image, the presence of MBs was also investigated. White matter hyperintensity severity was classified as confluent or not according to the description of imaging findings, figures of brain MRIs published in the literature, or direct observation of brain MRIs by the first author (MU). We further investigated the pathological findings associated with *HTRA1*-related CSVD. Patients were sorted into two groups (symptomatic carriers vs. CARASIL), and the clinical features and findings associated with each were compared.

### Statistical Analyses

Statistical analyses were performed using MATLAB R2018a software (9.4.0813654) (MathWorks, Inc., Natick, MA, USA). Continuous variables such as age at diagnosis were compared using Wilcoxon rank sum tests because of their non-normal distribution and non-equal variance. The Fisher exact test was used to compare the frequencies of variables such as vascular risk factors or neurological symptoms/signs. Statistical significance was defined as *p* < 0.05. If the information was not available, we excluded those data to perform statistical analysis.

## Results

### *HTRA1* Mutations in Symptomatic Carriers and CARASIL

*HTRA1* mutations identified are summarized in Table 1. Five mutations in symptomatic carriers (S121R, A123S, R133G, S284G, and D450H) had normal protease activity *in vitro* (18), and two mutations, S136G and Q151K, were non-pathogenic according to several *in silico* analyses. Given this, we excluded these seven mutations from further analyses (Supplementary Table 1). Forty-six patients were heterozygous symptomatic carriers of *HTRA1*-related CSVD, and the other 28 patients had CARASIL.

**Table 1:** Summary of mutations identified in patients with *HTRA1*-related CSVD.

**No**.	**cDNA**	**Amino acids**	**Domain**	**Trimerization**	**Protease activity**	**Patients**	**Independent families**	**Allele frequency of ExAC**	**PolyPhen2**	**SIFT**	**PROVEAN**	**PANTHER**	**References**
	**Symptomatic carriers**												
1	359G>A	G120D	Kazal-like	NA	Decreased	1	1	NA	Probably damaging	Tolerated	Deleterious	Deleterious	(26)
2	451C>T	Q151X	Kazal-like	NFM	NFM	1	1	NA					(25)
3	497G>T	R166L	Linker	Defective	Decreased	3	1	NA	Probably damaging	Deleterious	Deleterious	Deleterious	(6)
4	517G>C	A173P	Linker	Defective	Decreased	1	1	NA	Probably damaging	Deleterious	Deleterious	Deleterious	(6)
5	523G>A	V175M	Linker	NA	NA	2	1	0.000008236	Probably damaging	Deleterious	Deleterious	Deleterious	(9)
6	527T>C	V176A	Linker	NA	NA	1	1	NA	Probably damaging	Tolerated	Deleterious	Deleterious	(30)
7	536T>A	I179N	Linker	NA	Decreased	2	1	NA	Probably damaging	Deleterious	Deleterious	Deleterious	(26)
8	543delT	A182fs	Linker	NFM	NFM	1	1	NA					(26)
9	589C>T	R197X	Linker	NFM	NFM	1	1	NA					(30)
10	NA	G206E	Not L3/LD	NA	NA	1	1	NA	Probably damaging	Deleterious	Deleterious	Deleterious	(9)
11	646G>A	V216M	Not L3/LD	NA	NA	1	1	0.00001647	Probably damaging	Deleterious	Deleterious	neutral	(28)
12	767T>C	I256T	Not L3/LD	NA	Decreased	1	1	0.000008301	Probably damaging	Deleterious	Deleterious	Deleterious	(26)
13	827G>C	G276A	Not L3/LD	NA	Decreased	1	1	NA	Probably damaging	Deleterious	Deleterious	Deleterious	(26)
14	848G>A	G283E	LD	Defective	Decreased	1	1	NA	Probably damaging	Deleterious	Deleterious	Deleterious	(7)
15	851G>A	S284N	LD	NA	NA	1	1	NA	Probably damaging	Tolerated	Deleterious	Deleterious	(28)
16	852C>A	S284R	LD	Trimer	Decreased	1	1	NA	Probably damaging	Deleterious	Deleterious	Deleterious	(6)
17	854C>A	P285Q	LD	Trimer	Decreased	1	1	NA	Probably damaging	Deleterious	Deleterious	Deleterious	(6)
18	856T>G	F286V	LD	Trimer	Decreased	1	1	NA	Probably damaging	Deleterious	Deleterious	neutral	(6)
19	865C>T	Q289X	LD	NFM	NFM	2	1	NA					(26)
20	905G>A	R302Q	L3	Trimer	Decreased	5	3	NA	Probably damaging	Deleterious	Deleterious	Deleterious	(7, 27)
21	956C>T	T319I	Not L3/LD	Defective	Decreased	1	1	NA	Probably damaging	Tolerated	Deleterious	Deleterious	(7)
22	971A>C	N324T	Not L3/LD	NA	NA	1	1	NA	Probably damaging	Tolerated	Deleterious	Deleterious	(26)
23	973-1G>A	-	Not L3/LD	Splice site abnormalities	Splice site abnormalities	1	1	NA					(6)
	**CARASIL**												
1	126delG	E42fs	IGFBP	NFM	NFM	1	1	NA					(4)
2	161_162insAG	G56fs	IGFBP	NFM	NFM	1	1	NA					(14)
3	502A>T	K168X	Linker	NFM	NFM	1	1	NA					(3)
4	517G>A	A173T	Linker	Defective	Decreased	1	1	0.000008236	Probably damaging	Deleterious	Deleterious	Deleterious	(15)
5	616G>A	G206R	Not L3/LD	NA	NA	1	1	NA	Probably damaging	Deleterious	Deleterious	Deleterious	(5)
6	739delG	E247fs	Not L3/LD	NFM	NFM	1	1	NA					(3)
7	754G>A	A252T	Not L3/LD	Trimer	Decreased	1	1	0.000008258	Probably damaging	Deleterious	Deleterious	Deleterious	(1)
8	805insG	S270fs	Not L3/LD	NFM	NFM	3	1	NA					(17)
9	821G>A	R274Q	Not L3/LD	Defective	Decreased	2	1	0.000008266	Probably damaging	Tolerated	Deleterious	Deleterious	(11, 40)
10	830_831delAG	E277fs	Not L3/LD	NFM	NFM	1	1	NA					(3)
11	889G>A	V297M	Not L3/LD	Trimer	Decreased	2	2	NA	Probably damaging	Deleterious	Deleterious	Deleterious	(1)
12	958G>A	D320N	Not L3/LD	NA	NA	1	1	0.00002493	Probably damaging	Deleterious	Deleterious	Deleterious	(16)
13	961G>A	A321T	Not L3/LD	Trimer	Decreased	1	1	0.00003327	Probably damaging	Deleterious	Deleterious	Deleterious	(4)
14	983C>A	S328X	Not L3/LD	NFM	NFM	1	1	NA					(32)
15	1005+1G>T	-	Not L3/LD	Splice site abnormalities	Splice site abnormalities	1	1	NA					(29)
16	1021G>A	G341R	Not L3/LD	NA	NA	1	1	NA	Probably damaging	Deleterious	Deleterious	Deleterious	(16)
17	1091T>C	L364P	Not L3/LD	Trimer	Decreased	2	1	NA	Probably damaging	Deleterious	Deleterious	Deleterious	(23)
	**Both**												
1	496C>T	R166C	Linker	Defective	Decreased	8	4	NA	Probably damaging	Deleterious	Deleterious	Deleterious	(10, 13, 19, 21)
2	854C>T	P285L	LD	Trimer	Decreased	3	3	NA	Probably damaging	Deleterious	Deleterious	Deleterious	(2, 7)
3	883G>A	G295R	Not L3/LD	Defective	Decreased	5	2	0.000008258	Probably damaging	Deleterious	Deleterious	Deleterious	(9, 20)
4	904C>T	R302X	L3	NFM	NFM	3	3	NA					(1, 8)
5	1108C>T	R370X	PDZ	NMD	NMD	3	3	0.000008243					(1, 12, 24)

*Symptomatic carriers, heterozygous HTRA1-related CSVD; CARASIL, cerebral autosomal recessive arteriopathy with subcortical infarcts and leukoencephalopathy; NFM, nonsense/frameshift mutation; NMD, nonsense mediated decay; NA, not available; ExAC, Exome Aggregation Consortium; IGFBP, insulin-like growth factor binding domain; LD, loop D; L3, loop 3. The protease activity and trimerization represented in this table were referenced previously (7, 18, 26)*.

Overall, 30 missense, seven nonsense, six frameshift, and two splicing site mutations were enrolled. Twenty-one missense and seven truncated mutations (five nonsense, one frameshift, and one splicing site mutations) were identified in symptomatic carriers, whereas 12 missense and 10 truncated mutations (four nonsense, five frameshift, and one splicing site mutations) were identified in CARASIL. In symptomatic carriers, multiple independent families were reported in three mutations (R166C, P285L, and R302Q). Five mutations were identified both in CARASIL and symptomatic carriers (R166C, P285L, G295R, R302X, and R370X). Thus, among those mutations identified in symptomatic carriers and CARASIL, 70.0% of missense mutations and 46.7% of truncated mutations were identified in symptomatic carriers.

### Clustering Pathogenic *HTRA1* Mutations in Linker and Protease Domains

**Figure 1:**
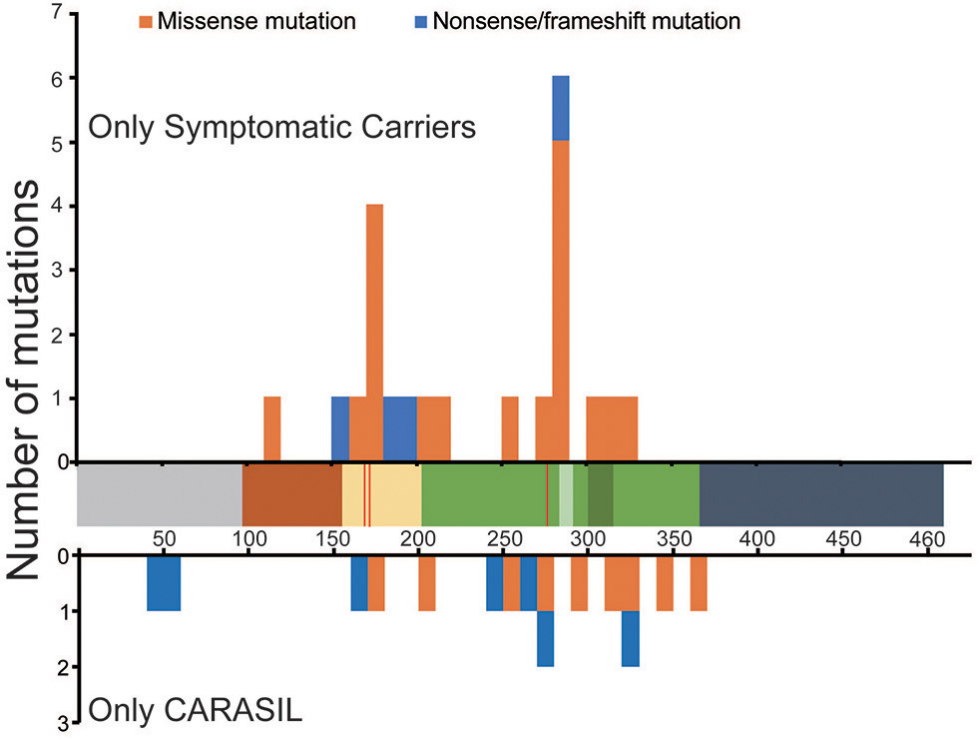
Location and frequency of mutations found in *HTRA1* genes. Distribution of *HTRA1* mutations. The number of mutations per 10 amino acids is shown. The upper bar graph indicates the mutations observed only in symptomatic carriers. The lower bar graph indicates the mutations observed in CARASIL patients alone. The horizontal axis shows the number of amino acids (AAs) in HTRA1 protein. The residues, which are important for trimerization, are indicated by the red line. Each colored box represents a functional domain, which are colored as follows: gray [N-terminus (1–98 AA)], brown [Kazal-like domain (99–157AA)], yellow [linker region(158–203 AA)], green [protease domain (204–364 AA)], light green [LD loop (283–291 AA), dark green [L3 loop (301–314 AA)], and navy blue [PDZ region (365–467 AA)]. The blue bar represents nonsense or frameshift mutations and the orange bar represents missense mutations.

**Figure 2:**
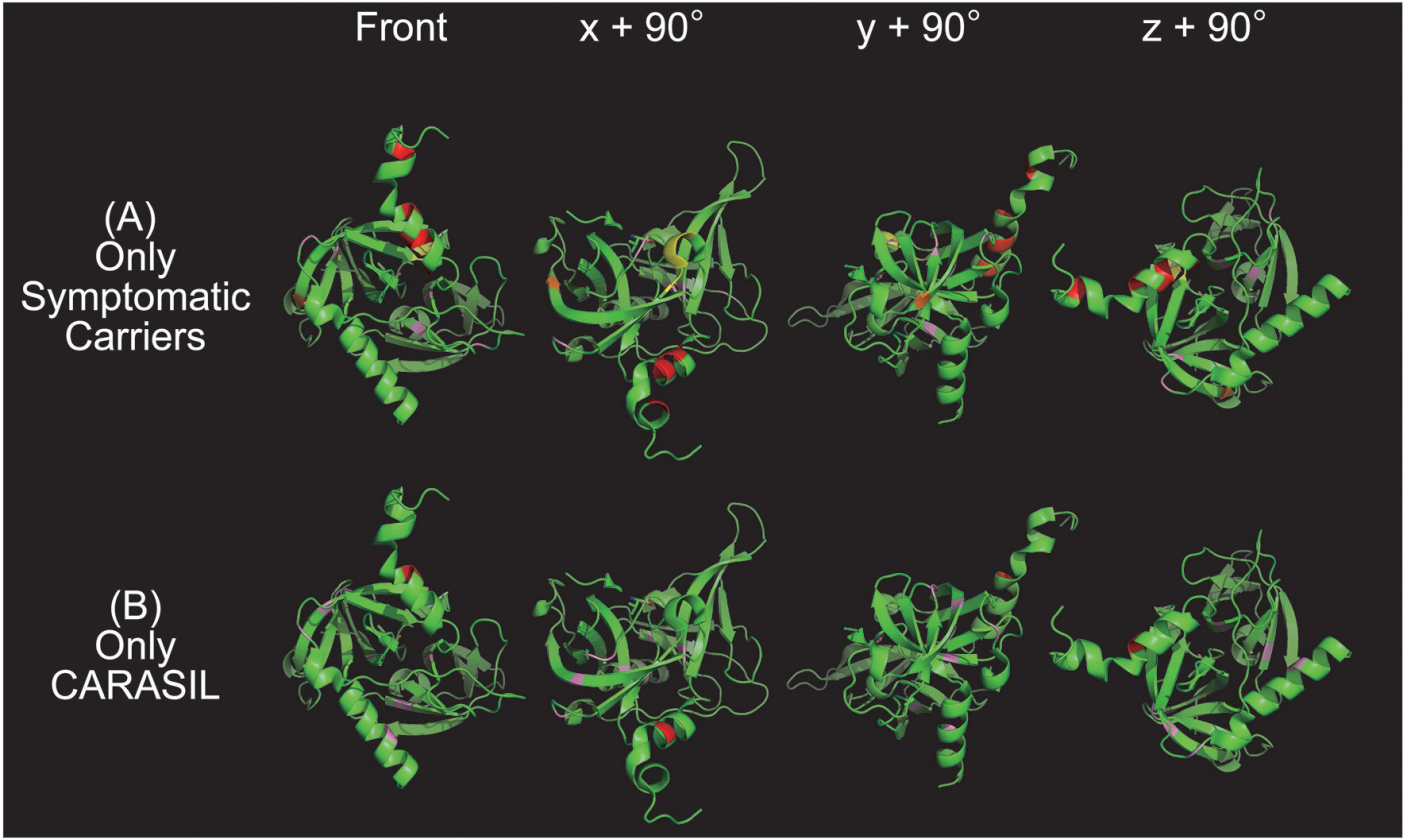
Color map of missense mutations identified in one group only. Three-dimensional HTRA1 monomer structures generated by PyMol are depicted. HTRA1 monomers are shown as green ribbons. The HTRA1 structure reference data set from the Research Collaboratory for Structural Bioinformatics (RCSB) Protein Data Bank (PDB ID: 3NZI) was used. Mutations in the linker region, protease domain (excluding the L3/LD loop), LD loop (amino acids 283 to 291), and L3 domain (amino acids 301 to 314) are highlighted in red, pink, yellow, and orange, respectively. The upper panel **(A)** and lower panel **(B)** show the missense mutations that were identified only in symptomatic carriers or CARASIL, respectively. On the left side are images from the front view and *x* + 90°, *y* + 90°, and *z* + 90° indicate the degree of rotation along the *x, y*, and *z* axes, respectively.

The locations of mutations only in symptomatic carriers or in CARASIL are summarized in Table 2. Figures 1, 2 show the location of mutations identified in each group. All missense mutations were located in linker or protease domains except for G120D, which located at Kazal-like domain. In symptomatic carriers, ~50% of the missense mutations were concentrated in two regions: from 166 to 179 (the linker region, including key residues necessary for the trimerization of *HTRA1*) and from 283 to 286 (the LD loop, which is important for *HTRA1* activation) (Table 1, Figure 1). In CARASIL, only one missense mutation was located within 166–179, and no mutations were located on the LD/L3 loop (Figure 1, Table 2). Eight of the nine missense mutations were dispersed throughout the protease domain. In both symptomatic carriers and CARASIL, nonsense or frameshift mutations were also predominantly located on linker or protease domains. In CARASIL, two frameshift mutations were located in the *IGFBP* domain. Nonsense/frameshift mutations were less frequent in symptomatic carriers than in patients with CARASIL (Table 2).

**Table 2:** Summary of *HTRA1* mutations identified in only one group.

**Items**	**Symptomatic carriers**	**CARASIL**	***p***
Total mutations	23	17	
Missense mutations	18 (78.3)	9 (52.9)	0.17
Kazal-like (99–157)	1 (4.3)	0	1.0
Linker region(158–203)	5 (21.7)	1 (5.9)	0.22
LD (283–291)/L3 (301–314)	6 (26.1)	0	0.03
Not L3/LD (204–364)	6 (26.1)	8 (47.1)	0.20
Nonsense/frameshift mutations	4 (17.4)	7 (41.2)	0.15
IGFBP (33–98)	0	2 (11.8)	0.17
Kazal-like (99–157)	1 (4.3)	0	1.0
Linker region(158–203)	2 (8.7)	1 (5.9)	1.0
LD (283–291)/L3 (301–314)	1 (4.3)	0	1.0
Not L3/LD (204–364)	0	4 (23.5)	0.03
Mutations in the splice site	1 (4.3)	1 (5.9)	1.0

*Symptomatic carriers, heterozygous HTRA1-related CSVD; CARASIL, cerebral autosomal recessive arteriopathy with subcortical infarcts and leukoencephalopathy*.

### Comparison of Symptomatic Carrier and CARASIL Clinical Features

Clinical and imaging findings from the included patients are summarized in Table 3. Twenty-eight mutations in 46 patients and 22 mutations in 28 patients were identified in symptomatic carriers and CARASIL, respectively.

**Table 3:** Summary of clinical and imaging information for patients with *HTRA1*-related CSVD.

	**Symptomatic carriers**	**CARASIL**	***p***
Patients	46	28	
**Age at the diagnosis**			
Means ± SD, years, (not reported)	59.8 ± 10.5, (0)	35.7 ± 8.8, (1)	<0.01
Range, years	31–78	24–53	
Male (%)	35 (76.1)	12 (42.9)	<0.01
**Family history**			
First or second relatives (%), (not reported)	40 (88.9), (1)	20 (74.1), (1)	0.112
Family history of parents (%), (not reported)	30 (75.0), (6)	7 (25.9), (1)	<0.01
-Cognitive impairments	9	0	
-Stroke	19	5	
-Leukoencephalopathy	4	2	
Consanguinity marriage of parents (%), (not reported)	0, (1)	21 (77.8), (1)	<0.01
**Risk factors**			
Hypertension (%), (not reported)	20 (45.5), (2)	0, (7)	<0.01
Diabetes mellitus (%), (not reported)	0, (13)	0, (9)	1.000
Dyslipidemia (%), (not reported)	7 (19.4), (10)	0, (11)	0.082
Alcohol (%), (not reported)	3 (9.7), (15)	1 (5.9), (11)	1.000
Smoking (%), (not reported)	8 (22.9), (11)	4 (23.5), (11)	1.000
**Age at the onset of neurological symptoms/signs**			
Means ± SD, years, (not reported)	54.1 ± 11.4, (6)	29.5 ± 5.5, (4)	<0.01
Range, years	29–77	20–40	
**Neurological symptoms/signs**			
Cognitive impairments (%), (not reported)	35 (77.8), (1)	22 (88.0), (3)	0.353
Gait disturbance (%), (not reported)	29 (67.4), (3)	25 (92.6), (1)	0.019
Episode of Stroke (%), (not reported)	29 (63.0), (0)	11 (40.7), (1)	0.089
-Ischemic stroke (%), (not reported)	28 (60.9), (0)	9 (34.6), (2)	0.049
-Hemorrhagic stroke (%), (not reported)	6 (15), (6)	1 (3.7), (1)	0.228
Any of cognitive impairments, gait disturbance and stroke (%)	41 (89.1)	28 (100.0)	0.150
Migraine (%), (not reported)	6 (35.3), (29)	2 (12.5), (12)	0.225
**Extraneurological symptoms/signs**			
Alopecia of younger onset (%), (not reported)	5 (13.2), (8)	24 (85.7), (0)	<0.01
Lumbago/spondylosis deformans (%), (not reported)	21 (60.0), (11)	30 (100.0), (0)	<0.01
**MRI findings**			
Confluent WMHs (%)	37 (81.3)	28 (100.0)	0.011
LIs (%), (not reported)	39 (97.5), (6)	24 (100.0), (4)	1.000
MBs (%), (not reported)	19 (73.1), (20)	11 (84.6), (15)	0.689

*Symptomatic carriers, heterozygous HTRA1-related CSVD; CARASIL, cerebral autosomal recessive arteriopathy with subcortical infarcts and leukoencephalopathy; WMHs, white matter hyperintensities; LIs, lacunar infarctions; MBs, microbleeds*.

The frequency of family history of first or second relatives was similar between groups (symptomatic carriers 88.9% vs. CARASIL 74.1%, *p* = 0.112). Five parents of CARASIL patients with R274Q, P285L, V297M, and R302X mutations had a history of stroke (1, 2, 11, 40–42). Furthermore, two parents of CARASIL patients who were carriers of E42fs (4) and G295R mutations (20) had leukoencephalopathy. Detailed family history of the parents of seven symptomatic carriers was not available (6, 26).

The frequency of male patients was significantly higher among the symptomatic carriers (76.1%) compared to the CARASIL patients (42.9%) (*p* < 0.01). The frequency of hypertension was also significantly higher in symptomatic carriers (45.5%) than CARASIL patients (0%) (*p* < 0.01).

Age at onset of neurological symptoms/signs was determined in 40 symptomatic carriers and 24 patients with CARASIL. The age at onset of neurological symptoms/signs was significantly higher in symptomatic carriers (54.0 ± 11.4 years) than in CARASIL patients (29.5 ± 5.5 years) (*p* < 0.01).

The youngest and oldest reported ages at onset of neurological symptoms/signs among symptomatic carriers were 29 (25) and 77 (30), respectively, and the youngest and oldest ages at onset of neurological symptoms/signs in CARASIL were 20 (29) and 40 (16), respectively. Patient age at diagnosis was significantly higher among symptomatic carriers (59.8 ± 10.5 years) than in CARASIL (35.7 ± 8.8 years) (*p* < 0.01).

There were also several differences in neurological symptoms/signs between the two groups. While the frequency of episode of stroke was more higher in symptomatic carriers (63.0%) than CARASIL (40.7%) (*p* = 0.089), gait disturbance was significantly less frequent in symptomatic carriers (67.4%) than CARASIL (92.6%) (*p* = 0.019). Notably, five symptomatic carriers did not exhibit neurological symptoms/signs at the time of diagnosis (6, 9, 24). The frequency both of alopecia of younger onset and lumbago/spondylosis deformans was significantly higher in CARASIL than symptomatic carriers (alopecia of younger onset: CARASIL 85.7% vs. symptomatic carriers 13.2%, *p* < 0.01; lumbago/spondylosis deformans: CARASIL 100% vs. symptomatic carriers 60.0%, *p* < 0.01). Other clinical findings, including recurrent rhinitis, have been reported in three patients with CARASIL (17). On brain MRIs, the frequency of confluent WMHs was significantly higher in CARASIL patients than symptomatic carriers (CARASIL 100% vs. symptomatic carriers 81.3%, *p* = 0.011). Frequency of LIs and MBs was similar between the two groups.

### Pathological Findings

Pathological findings for patients with *HTRA1*-related CSVD (two symptomatic carriers and four with CARASIL) are summarized in Table 4 (1, 7, 15, 31, 43–46). All patients, besides one Pakistani patient, were Japanese (15). Cardinal pathological features included extensive loss of medial smooth muscle cells, intimal proliferation, and splitting of the internal elastic lamina in the pial arteries, perforating arteries, and arterioles. Accumulation of TGF-β1 in the media was found in one symptomatic carrier and one CARASIL patient via immunohistochemistry (1, 44). In addition, fibronectin or extradomain A fibronectin, versican, and hyaluron were also positive (1, 46). In contrast, immunostaining for collagen types I, III, and IV was reduced in the adventitia (44). In four cases with CARASIL, the skin arteries had similar pathological findings, which included intimal proliferation or loss of smooth muscle cells in the small arteries (14, 23, 25, 41).

**Table 4:** Summary of pathological findings in *HTRA1*-related cerebral small vessel disease cases.

	**Symptomatic carriers**	**Symptomatic carriers**	**CARASIL**	**CARASIL**	**CARASIL**	**CARASIL**
Amino acids	p.G283E	p.R302Q	p.A173T	p.A252T	p.R302X	p.R302X
Sex	Male	Male	Female	Female	Male	Female
Age at pathological analysis	56	61	35	51	54	46
**Affected intracranial arteries**						
Large artery	NA	NA	NA	NA	+	+
Meningeal to leptomeningeal artery	NA	+	NA	+	+	+
Arterioles	+	+	+	+	+	+
Minimum diameter of arterioles (μm)	~40	<100	NA	NA	45	NA
Capillaries	NA	NA	NA	NA	NA	NA
**Pathological findings of affected intracranial arteries**						
Myointimal thickening	+	+	+	+	+	+
Multilayered elastic laminae	+	+	+	+	+	+
Hyalinosis	+	+	NA	+	+	NA
Loss of medial smooth muscle cell (SMC)	NA	+	+	+	+	+
Narrowing of vascular lumens	NA	+	NA	+	NA	+
**Positive findings with immunohistochemistry**						
Transforming growth factor	NA	+	NA	NA	NA	+
Phosphorylated Smad2	NA	NA	NA	NA	NA	+
Latency-associated peptide	NA	NA	NA	NA	NA	+
Extradomain A fibronectin	NA	NA	NA	NA	NA	+
Fibronectin	NA	NA	NA	+	NA	NA
Versican	NA	NA	NA	NA	NA	+
Hyaluronan	NA	NA	NA	NA	NA	+
Collagen type I	NA	NA	NA	Weak	NA	NA
Collagen type III	NA	NA	NA	Weak	NA	NA
Collagen type IV	NA	NA	NA	Weak	NA	NA
Electron microscopy	NA	+	NA	+	NA	NA
Findings		Dense deposit		Lysosome-like body		
				Lipofuscin-like body		
References	(7)	(43)	(15)	(31, 44, 45)	(44)	(1, 44, 46)

*Symptomatic carriers, heterozygous HTRA1-related CSVD; CARASIL, cerebral autosomal recessive arteriopathy with subcortical infarcts and leukoencephalopathy; NA, not available*.

Although there was no granular osmophilic material found in *HTRA1*-related CSVD samples, a characteristic findings of autosomal dominant cerebral arteriopathy with subcortical infarcts and leukoencephalopathy (CADASIL), electron microscopy did reveal some deposits in one CARASIL patient and in one symptomatic carrier (31, 43). Electron-dense deposits were also found in the cytoplasm of smooth muscles cells in CARASIL patients (31) and in the outer layer of the elastic lamina in symptomatic carriers (43).

## Discussion

In the present review, we have reconfirmed that symptomatic carriers have milder phenotypes than CARASIL patients. The symptomatic carriers show the elderly onset, lower frequency of extraneurological complications, and milder WMHs compared to those in CARASIL. Furthermore, even in cases with five mutations (R166C, P285L, G295R, R302X, and R370X), in which both CARASIL and symptomatic carriers have been reported, the symptomatic carriers showed a milder clinical phenotype. These results indicate that residual protease activity of HTRA1 is associated with clinical phenotype.

For the molecular mechanism of symptomatic carriers, we can speculate that the reducing residual HTRA1 activity may increase the risk of CSVD. First, we will discuss the mechanism of haploinsufficiency for the molecular pathogenesis of symptomatic carriers. Although initially no symptomatic carriers were demonstrated to have nonsense or frameshift mutations, five nonsense mutations (Q151X, R197X, Q289X, R302X, and R370X) and one frameshift mutation (A182fs) were identified in symptomatic carriers. Mutant HTRA1 protein expressed by some of these mutant alleles might exert a dominant-negative effect (26). However, in many cases, HTRA1 from the mutant alleles loses its activity because of the lack of an active site. Alternatively, HTRA1 protein expression from the mutant allele is reduced in quantity because of nonsense-mediated degradation of mRNA or unstable protein (1, 17). Therefore, we consider the haploinsufficiency theory as the underlying molecular mechanism in symptomatic carriers due to these mutations.

**Figure 3:**
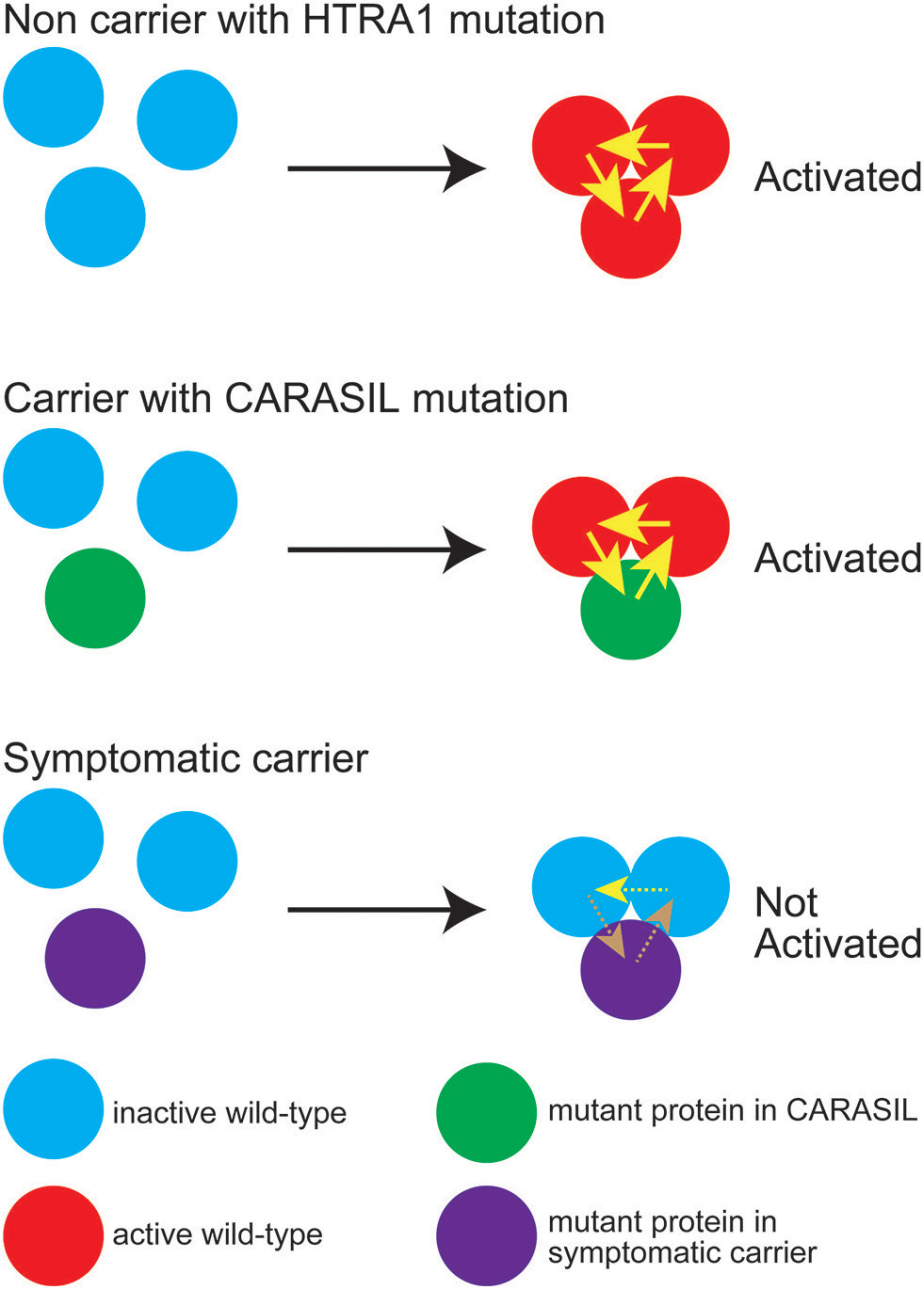
Diagram representing *HTRA1* protein activation status. The wild-type (WT) HTRA1 protein physiologically forms a trimer and activates the neighborhood HTRA1 protein through LD and the L3 domain (upper panel). Yellow arrows indicate activation of the neighboring HTRA1s through LD and the L3 domain. In carriers with CARASIL mutations, WT HTRA1, and mutant HTRA1 form a trimer and activate neighboring WT HTRA1 (middle panel). In symptomatic carriers, mutant HTRA1 interferes with the activation cascade of the trimer, which results in decreased WT HTRA1 protease activity (lower panel). Brown dashed arrows indicate failed activation of neighboring HTRA1s. It is still remained unknown whether WT protein can activate neighboring WT protein even in the trimeric state with dominant-negative mutant protein (yellow dashed line).

On the other hand, with respect to missense mutations, some mutations are found in the symptomatic carriers, and others are not. We have shown that the mutant HTRA1s, which are found in symptomatic carriers, are characterized by either an inability to form a trimer or a mutation in the L3/LD domain (18). HTRA1 activity is regulated by an allosteric mechanism in which monomers relay an activation signal to each other. Peptide binding serves as the allosteric activation signal, which is transmitted to the protease domain via the L3 sensor loop. L3 then transmits this signal to the activation domain of the neighboring subunit through an interaction with LD (33, 34). Thus, trimerization capacity and the L3 and LD loops both play an essential role in HTRA1 activation. In symptomatic carriers with missense mutations, failure of the HTRA1 activation cascade results in a lack of normal activation of WT HTRA1; that is, a dominant negative effect is elicited (Figure 3) (7).

Indeed, we found that the missense mutations observed in symptomatic carriers are concentrated around Y169 and F171 in the linker region or on the LD loop. Ring stacking interactions between Y169, F171, and F278 stabilize the HTRA1 trimer (34, 47). Therefore, mutations in this region disturb the formation of the trimer and decrease HTRA1 protease activity (7, 18). Moreover, we have revealed that some mutations such as G283E, which occur outside the ring-stacking region, result in trimerization failure, which consequently decrease WT protease activity (7, 18). In addition, none of the mutations observed only in CARASIL patients were located on the LD/L3 loop (Table 2). Differences in the distribution and properties of missense mutations between symptomatic carriers and CARASIL may explain why most parents of CARASIL patients did not exhibit symptoms/signs of CSVD.

We discuss the association between gene mutations and prevalent carriers. No symptomatic carriers have been reported in approximately half of the nonsense or frameshift mutations. Many of the prevalent carriers with missense mutations are also sporadic. In addition, the age at onset and severity of the disease are extremely divergent, even for the same mutation. The age at onset of neurological symptoms/signs in symptomatic carriers was widely distributed. Four symptomatic carriers exhibited neurological symptoms/signs before the age of 40 years (7, 10, 25, 27), whereas the oldest age at onset was 77 years (30). Furthermore, one symptomatic carrier did not present with any apparent neurological symptoms/signs despite being older than 70 years (24). In addition, heterogeneity of age and severity at onset have previously been reported in patients from the same family (19, 21). These indicate that mutations alone cannot explain the age at onset or the severity of the disease.

Thus, other factors may be involved in the onset and severity of CSVD in symptomatic carriers. We have reported that symptomatic carriers are more common in males and have vascular risk factors more frequently than CARASIL patients (7). The results suggest that gender and environmental factors may be involved in the development of CSVD, whereas strict control of vascular risk factors may intervene in the development of CSVD among patients with heterozygous mutations in *HTRA1*.

Limitations of the present study were as follows. First, the pathogenicity of several missense mutations has been undetermined. We excluded seven missense mutations according to the results of protease activity or *in silico* analysis. These mutations were assumed to be incidentally identified among carriers because of the preserved function. However, recent studies have shown that some missense mutations possibly influence the stability of mutant HTRA1 proteins (6, 48). This effect of the mutant protein might influence the residual protease activity. Second, our data primarily comprised the information and the description in each reported article. Especially, for symptomatic carriers, there is a possibility that some clinical information was underreported. Thus, some clinical features or imaging data in the present study have a lower frequency than those of the real clinical data. Third, we could not analyze the detailed distribution of WMHs in brain MRIs because of the lack of detailed descriptions or figures in several articles. Several characteristic findings of brain MRI have been reported in CARASIL such as anterior temporal lesions or arc sign (49), however, the frequencies of these findings remained to be unknown both in CARASIL and symptomatic carriers to date. Further research is required to clarify the specific findings in *HTRA1*-related CSVD. Fourth, no comparative study has assessed the pathological findings between the two groups. Hence, several significant problems remain unresolved, such as the severity and distribution of the diseased vessels in symptomatic carriers as compared with those in patients with CARASIL. Additional research is required in the future to analyze the difference of pathological features between symptomatic carriers and patients with CARASIL.

The results described here support careful counseling of *HTRA1* mutation carriers by genetic counselors, who should consider the differential pathogenicity of the various *HTRA1* variants identified here. Eighteen of the twenty-eight mutations found in symptomatic carriers have only been reported in a single case each. This reinforces the notion that mutation carriers do not always develop CSVD; rather, these mutations appear to serve as a risk factor for CSVD. CADASIL type 2 (OMIM 616779) has been proposed as a name for symptomatic *HTRA1* mutation carriers. However, the penetrance of many of the mutations identified in symptomatic carriers appears to be low. Therefore, it may not be suitable to include “dominant” in any name for this condition. Instead, *HTRA1*-related CSVD, which includes both symptomatic carriers and CARASIL patients, may serve as an appropriate name for this condition. Further research is required to elucidate the pathogenicity of each *HTRA1* mutation in the development of CSVD.

## Conclusion

In the present article, we conducted a literature review of *HTRA1*-related CSVD. We found that the clinical symptoms/signs of symptomatic carriers were milder than those of CARASIL patients, a result that was supported by prior work. The locations of mutations found in symptomatic carriers also differed from those found in CARASIL patients. Missense mutations in symptomatic carriers were frequently located in the linker region or L3/LD domain, whereas missense mutations in CARASIL patients were more frequently located in the protease domain and rarely in the L3/LD domain. Both the linker region and L3/LD domain are critical sites for HTRA1 protein activation via intermolecular communication mechanisms. Mutations in the linker region or L3/LD domain will interfere with this activation, which has a dominant negative effect, whereas heterozygous *HTRA1* mutations, which are located outside the linker or L3/LD domain, require a careful evaluation of pathogenicity. The findings presented here will improve genetic counseling for both the relatives of CARASIL patients and carriers of *HTRA1* variants with sporadic CSVD.

## Author Contributions

MU drafted the manuscript, devised the study concept and design, collected data, and performed analyses. HN revised the manuscript, interpreted the data, and supervised the study. NS, SA, MK, TI, and TM revised the manuscript and interpreted the data. AK and TK acquired and interpreted data. NH, YN, KP, AN, AH, DK, ASa, ASh, ME-G, AA, MY, and IM acquired clinical data. OO revised the manuscript, devised the study concept and design, and supervised the study. All authors contributed to the article and approved the submitted version.

## Conflict of Interest

OO has received speaking honoraria from Kyowa Hakko Kirin Co., Ltd., Bristol-Myers Squibb, Ono Pharmaceutical Co., Ltd., Mitsubishi Tanabe Pharm, Takeda, Daiichi-Sankyo, FUJIFILM, SANOFI, and FP-pharm. The remaining authors declare that the research was conducted in the absence of any commercial or financial relationships that could be construed as a potential conflict of interest.

## Footnotes

^1^ http://exac.broadinstitute.org/

^2^ http://pford.info/vapros/

^3^ http://www.rcsb.org/pdb/home/home.do

^4^ https://github.com/schrodinger/pymol-open-source
