# Supplementary material for: HTRA1-Related Cerebral Small Vessel Disease: A Review of the Literature
Source: Front Neurol. 2020 Jul 3;11:545. doi: 10.3389/fneur.2020.00545 (PMC7351529; doi:10.3389/fneur.2020.00545)
Supplement: Supplementary file 1 [file Table_1.DOCX]

| No | cDNA | Amino Acids | Domain | Trimerization | Protease activity | Patients | Independent  families | Allele frequency of ExAC | PolyPhen2 | SIFT | PROVEAN | PANTHER | References |
| --- | --- | --- | --- | --- | --- | --- | --- | --- | --- | --- | --- | --- | --- |
|  | Symptomatic Carriers |  |  |  |  |  |  |  |  |  |  |  |  |
| 1 | 361A>C | S121R | Kazal-like | NA | Normal | 1 | 1 | NA | probably damaging | DELETERIOUS | Deleterious | Deleterious | (1) |
| 2 | 367G>T | A123S | Kazal-like | NA | Increased | 1 | 1 | NA | possibly damaging | TOLERATED | neutral | neutral | (1) |
| 3 | 397C>G | R133G | Kazal-like | NA | Increased | 1 | 1 | NA | possibly damaging | TOLERATED | Deleterious | neutral | (1) |
| 4 | NA | S136G | Kazal-like | NA | NA | 1 | 1 | NA | benign | TOLERATED | Deleterious | Deleterious | (2) |
| 5 | 451C>A | Q151K | Kazal-like | NA | NA | 2 | 2 | 0.0001868 | possibly damaging | TOLERATED | Deleterious | neutral | (2, 3) |
| 6 | 850A>G | S284G | LD | NA | Normal | 1 | 1 | NA | probably damaging | DELETERIOUS | Deleterious | Deleterious | (1) |
| 7 | 1348G>C | D450H | PDZ | NA | Normal | 1 | 1 | NA | possibly damaging | DELETERIOUS | Deleterious | neutral | (1) |

Supplementary Table.

Table 1. Summary of excluded mutations identified in patients with *HTRA1*-related CSVD.
symptomatic carriers = heterozygous *HTRA1*-related CSVD, NA = not available. ExAC = Exome Aggregation Consortium. LD = loop D.

**References**

1 Verdura E., Herve D., Scharrer E., Amador Mdel M., Guyant-Marechal L., Philippi A., et al. (2015). Heterozygous *HTRA1* mutations are associated with autosomal dominant cerebral small vessel disease. Brain 138, 2347-58.

2 Di Donato I., Bianchi S., Gallus G.N., Cerase A., Taglia I., Pescini F., et al. (2017). Heterozygous mutations of *HTRA1* gene in patients with familial cerebral small vessel disease. CNS Neurosci Ther 23, 759-765.

3 Pati A.R., Battisti C., Taglia I., Galluzzi P., Bianchi M., and Federico A. (2018). A new case of autosomal dominant small vessel disease carrying a novel heterozygous mutation in *HTRA1* gene: 2-year follow-up. Neurol Sci 39, 1479-1481.
